# The Drain Dilemma: A Systematic Review and Meta-Analysis of Drain-Free Abdominal Closure With Progressive Tension Sutures Against Drain-Assisted Closure for Abdominal Flaps in Breast Reconstruction

**DOI:** 10.7759/cureus.18924

**Published:** 2021-10-20

**Authors:** Abbas Ali Khan, Benjamin Wood, Zabihullah Abdul, Shafiq Rahman, Ammar Allouni

**Affiliations:** 1 Cardiology, Royal Preston Hospital, Preston, GBR; 2 Plastic Surgery, Sheffield Teaching Hospitals NHS Foundation Trust, Northern General Hospital, Sheffield, GBR; 3 Plastic Surgery, Bradford Teaching Hospitals NHS Foundation Trust, Bradford Royal Infirmary, Bradford, GBR; 4 Plastic Surgery, Hull University Teaching Hospitals NHS Trust, Hull Royal Infirmary, Hull, GBR

**Keywords:** breast reconstruction, drain, abdominal flap, operative time, complication rate, duration of hospital stay

## Abstract

The use of abdominal drains in donor site closure following breast reconstruction with abdominal flaps is widespread. Our review aimed to compare the outcomes of donor site closure with and without the use of abdominal drains following breast reconstruction with abdominal flaps.

Randomized, non-randomized, and observational studies that compared the use of drains vs. no drain in breast reconstruction were included by searching MEDLINE, EMBASE, EMCARE, CINAHL, and the Cochrane Central Register of Controlled Trials (CENTRAL).

Four studies enrolling 327 participants were identified. A statistically significant difference was found in terms of duration of hospital stay favouring abdominal closure without the use of drains (MD = -1.15, 95% CI = -1.88 tom-0.42, P=0.002), with a similar difference found in terms of overall complication rate (OR = 0.44, 95% CI = 0.23 to 0.83, p=0.01). Likewise, a statistically significant difference was found favouring abdominal closure without the use of drains for the secondary outcome of operative time (MD = -55.95, 95% CI = -107.19 to -4.74, p=0.03).

Abdominal closure without drains following breast reconstructions with abdominal flaps is superior to closure with drains.

## Introduction and background

Free flap breast reconstruction is utilised for 14% of patients undergoing immediate breast reconstruction and 33% undergoing delayed reconstruction [1]. The use of the deep inferior epigastric perforator (DIEP) free flap for breast reconstruction is popular due to its supple aesthetically similar tissue, minimal donor site impact, and cost-effective nature [2-3].

Although used by 90% of plastic surgeons, the use of abdominal drains during donor site closure in breast reconstruction with abdominal flaps is widely debated across the surgical literature [1]. Inserting a drain is based on the premise that it has a role in reducing dead space by preventing fluid accumulation, thereby minimising subsequent complications such as seroma formation and wound dehiscence [4]. Drain use, however, does carry caveats to patient care, including an increased risk of infection, pain, reduced mobility, greater nursing requirements, as well as prolonged hospital admission [5].

**Figure 1:**
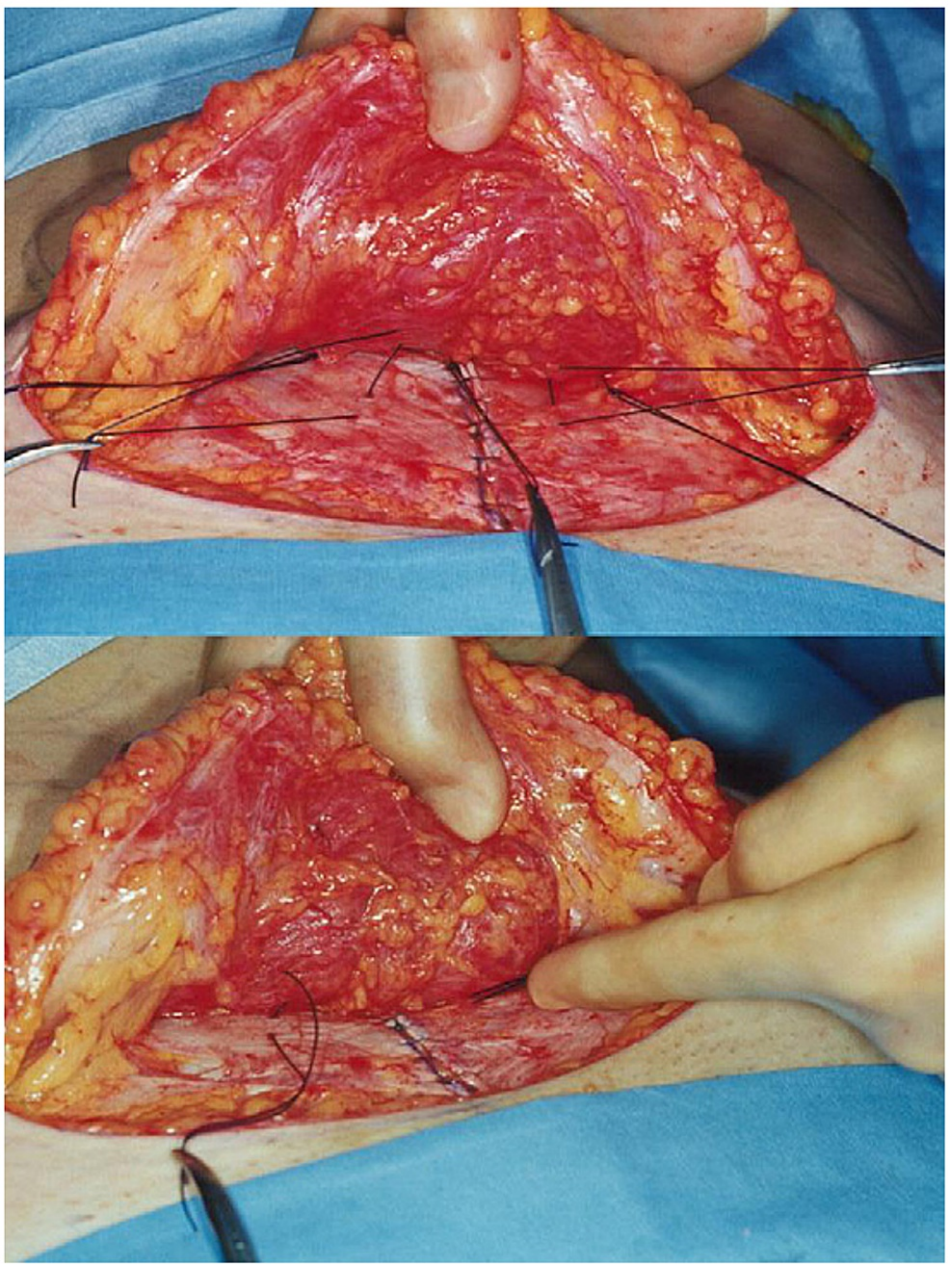
Progressive tension suture placement in no-drain abdominoplasty Reprinted from Clinics in Plastic Surgery, Vol. number 37, Pollock T, Pollock H, No-Drain Abdominoplasty With Progressive Tension Sutures, Page No. 519, Copyright (2010), with permission from Elsevier.

Taking this into account, alternative techniques have been used to circumvent the drawbacks associated with abdominal drains, including tissue sealants, progressive tension sutures (PTS), as well as barbed sutures [6]. PTS have been adapted as a reliable technique for dead space reduction post abdominal closure by evenly distributing tension across the surgical site and minimising shearing force [7]. Initially described by Pollock, and shown in Figure 1 below, PTS have demonstrated encouraging results with comparable complication rates to standard closure with drains [8-11]. Several recent reports in the literature have compared the two modalities, yet there is no study to amalgamate outcomes and provide the best available evidence to guide surgeons [4-5]. The authors, therefore, report the first systematic review and meta-analysis assessing the outcomes of drain-free closure of abdominal wounds in breast reconstruction.

## Review

Methodology

A systematic review and meta-analysis were performed as per Preferred Reporting Items for Systematic Reviews and Meta-Analyses (PRISMA) and Assessment of Multiple Systematic Reviews (AMSTAR) guidelines [12]. This study is registered with the Research Registry and the unique identifying number is reviewregistry1030.

Eligibility criteria

All randomised and non-randomised trials, as well as observational studies that compared the use of drains versus no drains in abdominal closure in the setting of breast reconstruction, were included. Patients were included regardless of age, sex, or comorbidity status. Articles not reported in English were excluded from the review.

Outcomes of interest

The primary outcome measures included length of hospital stay as well as the incidence of complications. The secondary outcome measure was operating time.

Literature search strategy

Two authors (AAK, SR, searched the following electronic databases: MEDLINE, EMBASE, EMCARE, CINAHL, and the Cochrane Central Register of Controlled Trials (CENTRAL). The last search was conducted on August 25, 2020. The following terminologies were employed: “drain”, “no drain”, “progressive tension sutures”, “PTS”, “abdominal closure”, “abdomen”, “free flap”, “breast reconstruction” and “breast surgery”. These were all adjoined with adjuncts of “and” as well as “or”.

Selection of studies 

Two authors (AAK, SR) independently assessed the title and abstract of all articles that met the eligibility criteria with any discrepancy being resolved by discussion with a third author (AA).

Data extraction and management

An electronic data extraction spreadsheet was drafted per Cochrane’s data collection form for intervention reviews. It underwent preliminary testing in random articles. The spreadsheet data was as follows; first author, year of publication, country of origin of the corresponding author, journal in which the study was published, study design, study size, the use of abdominal drain or not, operating time, cosmetic outcome, and incidence of postoperative complications.

Data synthesis

Review Manager 5.4 software ([Computer program], Version 5.4, The Cochrane Collaboration, 2020) was used to conduct the data analysis using a random-effects model, and results were reported in forest plots with 95% confidence intervals (CIs). For continuous outcome data, the mean difference (MD) was used to assess both groups, and dichotomous outcomes were analysed with an odds ratio.

Assessment of heterogeneity

Heterogeneity among the studies was assessed using the Cochran Q test (χ2) as well as the I 2 score, which was interpreted using the following reference: 0% to 25% was representative of low heterogeneity; 25% to 75% (moderate heterogeneity); and 75% to 100% (high heterogeneity).

Literature search

**Figure 2:**
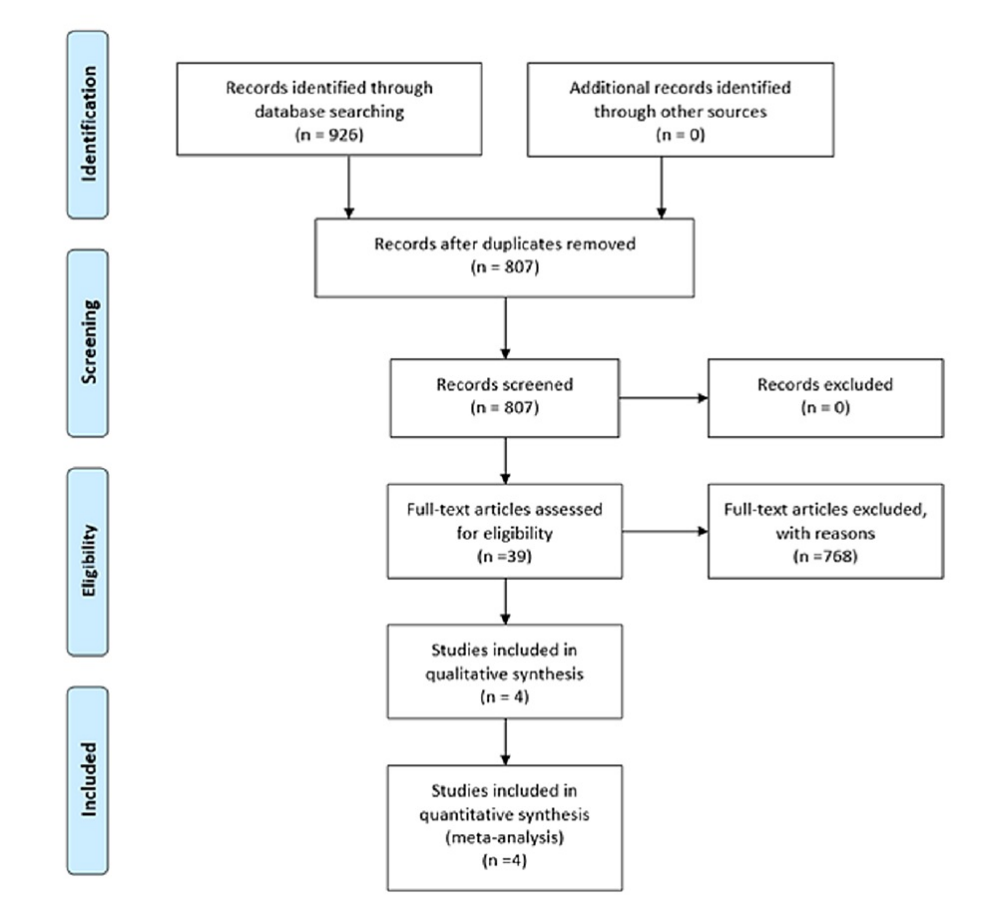
PRISMA flow diagram for article screening and selection PRISMA: Preferred Reporting Items for Systematic Reviews and Meta-Analyses

Our search strategy retrieved 19 studies and, after thorough screening, we identified four studies that met the eligibility criteria (Figure 2).

Description of Studies

Mohan et al.: They performed a single-centre retrospective review of 93 patients undergoing DIEP flap reconstructions [13]. Patients were divided according to the method of abdominal closure into standard abdominal closure with drains (51) and barbed progressive tension sutures without drains (42). There was no external source of funding.

Nagarkar et al.: They conducted a two-centre retrospective review of 75 patients undergoing DIEP flap reconstructions [5]. Patients were divided according to methods of abdominal closure. Twenty-five (25) patients underwent closure with barbed progressive tension sutures without a drain, 25 underwent closure with progressive tension sutures with an abdominal drain, and 25 underwent standard closure with an abdominal drain. There was no external source of funding.

Thacoor et al.: They undertook a single-centre retrospective review of patients undergoing DIEP flap reconstruction [4]. Patients were divided into groups based on the method of abdominal closure. Thirty-five (35) patients underwent drain-free abdominal closure (group A), 33 patients underwent closure with a drain with the drain removed on postoperative day 3 regardless of drain output (group b), and 41 patients underwent abdominal closure with a drain with the drain removed based on drain output (group c). For the purpose of analysis, groups b and c were combined. There was no external source of funding reported.

Chan et al.: They performed a single-centre retrospective review of 50 patients undergoing DIEP and transverse rectus abdominis (TRAM) flap reconstructions [14]. The TRAM flap reconstructions included muscle-sparing free TRAM (3), free TRAM (17), and pedicled TRAM flap (4). All patients were divided into groups depending on the method of closure. Twenty-five (25) patients underwent abdominal closure with barbed progressive tension sutures without an abdominal drain, and 25 patients underwent abdominal closure with a drain. There was no external source of funding reported.

The characteristics of the included studies are summarised in Table 1.

**Table 1:** Study characteristics

Study	Year	Type of breast reconstruction	Drain Group (n)	No drain group (n)	Method of abdominal closure in the drain group
Thacoor [4]	2018	Deep Inferior Epigastric Perforator Flap	74	35	3/0 PDS sutures to Scarpa’s fascia, 3/0 monocryl deep dermal sutures, running subcuticular 3/0 prolene skin suture
Nagarkar [5]	2016	Deep Inferior Epigastric Perforator Flap	50	25	Interrupted progressive tension sutures with abdominal drain (n=25)/Scarpa’s fascia reapproximated using 3 point suture, 3/0 vicryl deep dermal sutures, 2-0 VLOC 90 running dermal suture (n=25)
Mohan [13]	2015	Deep Inferior Epigastric Perforator Flap	51	42	Layered closure – PDS for Scarpa’s fascia, 3/0 monocryl for deep dermis, monoderm for skin. 2-4 drains.
Chan [14]	2019	Deep Inferior Epigastric Perforator/Transverse Rectus Abdominus Myocutaneous Flap (pedicled, free, free muscle-sparing TRAM)	25 (DIEP = 13, TRAM = 12)	25 (DIEP = 17, TRAM = 8)	Multi-layer closure 2/0 vicryl to Scarpa’s fascia, 3/0 vicryl for dermis, 3/0 monocryl for subcuticular closure.

Primary outcomes

Length of Hospital Stay

Length of hospital stay was assessed in terms of days taken from operation to discharge from hospital in patients undergoing abdominal closure following DIEP/TRAM reconstruction with and without drains. Mohan et al., Chan et al., and Thacoor et al. reported length of hospital stay in terms of days. Nagarkar et al. did not mention this.

**Figure 3:**
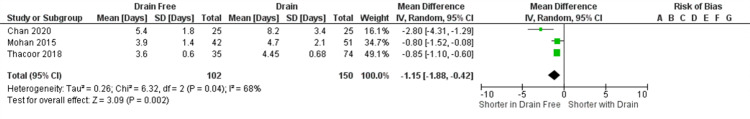
Length of hospital stay; forest plot comparing the length of hospital stay measured in days, favouring drain-free abdominal closure Based on data reported by Chan et al., Mohan et al. and Thacoor et al. [4,13-14] SD = standard deviation; IV = weighted mean difference; CI = confidence interval; Chi2 = chi-square statistic; p = p value; I2 = I-square heterogeneity statistic; Z = Z statistic

In Figure 3, length of hospital stay was reported in 102 patients from the above three studies. There was a statistically significant difference seen in the mean difference analysis, showing a shorter duration of stay in the drain-free group (MD = -1.15, CI = -1.88 to -0.42, p<0.002). Heterogeneity proved to be moderate with an I² value of 68% and P = 0.04.

Overall Complication Rate

The studies in our analysis all reported rates of seroma and dehiscence. Umbilical loss was reported by all but Thacoor et al., while haematoma formation was reported by all but Chan et al. Hypertrophic scarring was reported by Chan et al. alone.

**Figure 4:**
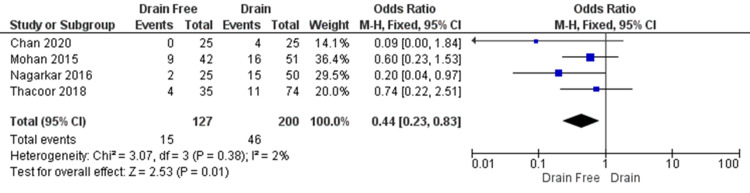
Overall complication rate; forest plot showing overall complication rate was lower in the drain-free abdominal closure group as reported across the four included studies Figures based on reports by Chan et al., Mohan et al., Nagargkar et al., and Thacoor et al. [4-5,13-14] CI = confidence interval; df = degrees of freedom; Chi2 = chi-square statistic; p = p value; I2 = I-square heterogeneity statistic; Z = Z statistic; M-H=Mantel-Haenszel

The overall complication rate was calculated by summating events across all types of complications from each study. It is not clear from the reports whether each complication occurred in unique patients or if several of the reported complications occurred in one patient. We have not sought further information to clarify this. Figure 4 shows the overall complication rate reported in 327 participants from all four studies. There was a statistically significant difference between the closure with abdominal drain group and the drain-free closure group, with complications less likely to occur in the latter (OR = 0.44, CI = 0.23 to 0.83, P = 0.01). A low level of heterogeneity was found (I² = 2%, P = 0.38).

Seroma

**Figure 5:**
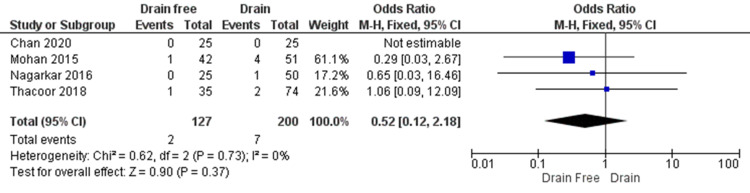
A comparison of the rate of seroma formation, with the outcome of seroma formation occurring less frequently in the drain-free abdominal closure group as reported by Thacoor et al., Nagarkar et al., and Mohan et al. Chan et al. did not encounter seroma formation in their patients. Data reported by Chan et al., Mohan et al., Nagargkar et al., and Thacoor et al. [4-5,13-14] CI = confidence interval; Chi2 = chi-square statistic; p = p value; I2 = I-square heterogeneity statistic; Z = Z statistic; M-H=Mantel-Haenszel

Seroma formation rate was reported in all four studies across 327 participants (Figure 5). No significant difference was seen between the groups (OR = 0.52, CI = 0.12 to 2.18, P = 0.37). There was a low level of heterogeneity across the studies. (I² = 0%, P = 0.73).

Dehiscence

**Figure 6:**
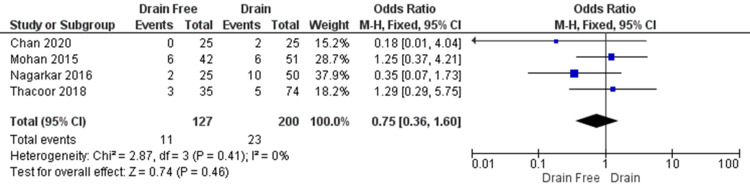
Wound dehiscence was reported in all four included studies. Mohan et al. and Thacoor et al. reported rates of wound dehiscence occurring less frequently in abdominal closure with drains, however, the overall rate of dehiscence was lower in the drain-free group. Data reported by Chan et al., Mohan et al., Nagarkar et al., and Thacoor et al. [4-5,13-14] CI = confidence interval; df = degrees of freedom; Chi2 = chi-square statistic; p = p value; I2 = I-square heterogeneity statistic; Z = Z statistic; M-H=Mantel-Haenszel

The rate of dehiscence was, likewise, reported in all four studied, including 327 participants (Figure 6). No significant difference was seen (OR = 0.75, CI = 0.36 to 1.6, P = 0.46). There a was a low level of heterogeneity observed. (I² = 0%, P = 0.41).

Haematoma

**Figure 7:**
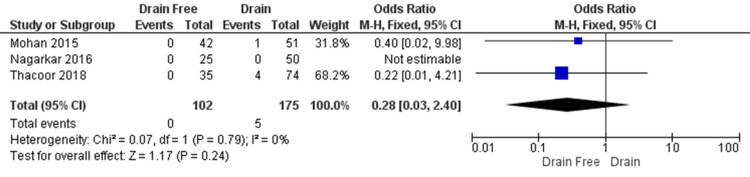
Haematoma formation; Nagarkar et al. did not encounter any haematoma formation amongst their participants. Overall, the rate of haematoma formation was lower in the drain free group. Data reported by Mohan et al., Nagarkar et al., and Thacoor et al. [4-5,13] CI = confidence interval; Chi2 = chi-square statistic; p = p value; I2 = I-square heterogeneity statistic; Z = Z statistic; M-H=Mantel-Haenszel

The rate of haematoma formation was reported in three studies (Mohan et al., Nagarkar et al., and Thacoor et al.) in 277 patients (Figure 7). No significant difference was seen between groups (OR = 0.28, CI = 0.03 to 2.40, P = 0.24). Heterogeneity was low across the studies included. (I² = 0%, P = 0.79).

Umbilical Loss

**Figure 8:**
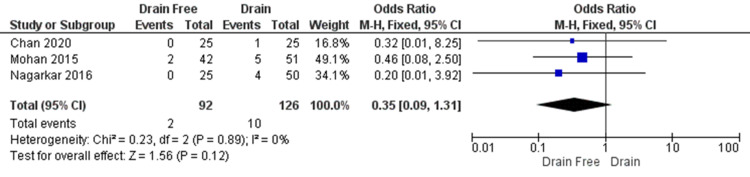
Umbilical loss was less likely to occur in drain free abdominal closure as was reported by Chan et al, Mohan et al and Nagarkar et al. Data reported by Chan et al, Mohan et al, and Nagarkar et al. [5,13-14] CI = confidence interval; df = degrees of freedom; Chi2 = chi-square statistic; p = p value; I2 = I-square heterogeneity statistic; Z = Z statistic; M-H=Mantel-Haenszel

The rate of umbilical loss was reported by Chan et al., Mohan et al., and Nagarkar et al. in a total of 218 participants (Figure 8). There was no significant difference seen between the groups. (OR = 0.35, CI = 0.09 to 1.31, P = 0.12). Heterogeneity was low (I² = 0%, P = 0.89).

Secondary outcomes

Operative Time

**Figure 9:**
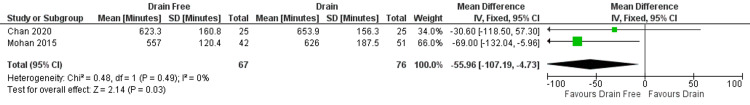
Operative time was reported in minutes by Chan et al. and Mohan et al. This was significantly lower in the drain-free group as depicted in this forest plot. Data reported by Chan et al. and Mohan et al. [13-14] SD = standard deviation; IV = weighted mean difference; CI = confidence interval; df = degrees of freedom; Chi2 = chi-square statistic; p = p value; I2 = I-square heterogeneity statistic; Z = Z statistic

Operative time was reported by Chan et al. and Mohan et al. in terms of minutes. This was reported across a total of 143 participants (Figure 9). A statistical difference was seen between the groups, with the operative time being less in the drain-free abdominal closure group (MD = -55.96, CI = -107.19 to -4.73, P = 0.03). Heterogeneity was low across the studies (I² = 0%, P = 0.49).

Methodological quality assessment

The quality of the included studies was assessed using the Newcastle-Ottawa scale. The summary is included in Table 2 below.

**Table 2:** Risk of bias assessment (Newcastle-Ottawa Scale)

	Selection				Comparability	Outcome			Quality Score
Study	Representativeness of the expose cohort	Selection of the non-exposed cohort from the same source as the exposed cohort	Ascertainment of exposure	An outcome of interest not present at the start of the study	Comparability of cohorts	Assessment of outcome	Follow up long enough for an outcome to occur	Adequacy of follow-up	Total
Thacoor [4]	Description of the derivation of the cohort not provided	Yes *	Secure records used (medical notes) *	Yes *	Cohorts were similar in terms of age and characteristics **	Reference to secure medical records *	Yes *	All subjects accounted for *	8
Nagarkar [5]	Description of the derivation of cohort not provided	Yes *	Secure records used (medical charts) *	Yes *	Cohorts were similar in terms of age and characteristics **	Reference to secure medical records *	Yes *	All subjects accounted for *	8
Mohan [13]	Description of the derivation of cohort not provided	Yes *	Secure records used (medical charts) *	Yes *	Cohorts were similar in terms of age and characteristics **	Reference to secure medical records *	Yes *	All subjects accounted for *	8
Chan [14]	Description of the derivation of cohort not provided	Yes *	Secure records used (medical charts) *	Yes *	Cohorts similar in terms of age *	Reference to secure medical records *	Yes *	All subjects accounted for *	7

Discussion

The analysis showed that abdominal closure following breast reconstruction using abdominal flaps without the use of a drain was superior to drain-assisted closure in terms of both primary and secondary outcome measures. Avoiding a drain has been found to significantly reduce both the duration of hospital stay (p= 0.002) and overall complication rates (p = 0.01). Furthermore, the operative time for abdominal closure was significantly shorter, with a mean difference of -55.96 minutes when compared to wound closure with a drain (p = 0.03).

Drain use remains common practice across the surgical field. There is, however, as seen in other surgical specialities, such as head and neck surgery, a scarcity of evidence to support or negate drain use post abdominal closure following breast reconstruction [15-16]. Furthermore, there is a paucity of evidence to guide drain management, time of removal, and antibiotic use [13]. This study has demonstrated that based on the currently available evidence, a drain does not significantly reduce complication rates of seroma, haematoma, or wound dehiscence when used in abdominal closure post-free-flap breast reconstruction; questioning the widely quoted reasoning for drain insertion in the first instance.

The results of this study are mirrored somewhat by a recent meta-analysis and systematic review by Li et al. (2020) who found that PTS without a drain has been shown to significantly reduce seroma rates in patients undergoing abdominoplasty [17]. Jabbour et al. (2016) further reported from a meta-analysis and systematic review that the use of a drain in addition to PTS did not affect seroma formation when compared to PTS alone thus again deliberating the need for surgical drains [7]. Like Miranda et al. (2014), the authors recognise that DIEP free-flap reconstruction does require further dissection and thus outcomes from abdominoplasty may not be directly transferable [1]. The general principles, however, of shearing and generalised inflammation will occur in DIEP surgery and thus the results from both Li et al. and Jabbour et al. do outline the positive outcomes achievable without drain use [7,17].

Progressive tension sutures that plicate the abdominoplasty flap to the abdominal wall have provided positive outcomes in breast reconstructive surgery since they were first adopted by Pollock and Pollock in 2000 [8]. With variations on the original technique, a variety of authors have demonstrated a reduction in postoperative complications. Nagarkar et al. found that the use of PTS when compared to PTS and drain yielded no significant difference in seroma formation while Liang et al. highlighted a reduced drain output when PTS was used and a shorter length of stay in a group closed with PTS only [5,18].

Furthermore, PTS is a useful closure technique when performing transverse rectus abdominis myocutaneous flap reconstruction. Rossetto et al. reported a 50% reduction in drain output when PTS was used alongside drains while Chan et al. reported that even when using PTS alone, it significantly lowered seroma rates and wound dehiscence and allowed for faster discharge and a more pleasing aesthetic outcome by strategic placement of PTS in recreating the linea alba and semiluminaris [14,19].

Mohan et al. advocate the use of barbed self-anchoring sutures and minimal lateral dissection of the abdominoplasty flap to optimise speed and minimise infection [13]. They also demonstrated that when compared to standard drain closure following DIEP breast reconstruction, barbed PTS offers a faster time to discharge, lower analgesic requirements and comparable seroma rates. Lower seroma rates have also been reflected by Landis et al. when using PTS as an adjunct in the closure of latissimus dorsi flaps for breast reconstruction, and if used in conjunction with a drain can expedite its postoperative removal [20]. This has furthered previous evidence from a review by Sajad et al. who showed the importance of quilting the latissimus dorsi donor site in minimizing complications [21]. Griner et al. have also demonstrated the effectiveness of PTS when applied in implant-based breast reconstruction following mastectomy with a reduction in seroma rates and less reliance on suction drains [22].

Preoperative optimisation before breast reconstruction cannot be overemphasised as highlighted by Cheng et al. 2015 [23]. Based on a 10-year retrospective review of 758 DIEP procedures, complications of fat necrosis were seen in 12.9%, seroma in 4.6%, haematoma in 1.8%, wound infection in 2.8%, partial flap loss in 2.5%, and total flap loss in 0.5%, with 5.9% of patients returning to the theatre for associated complications, with Gill et al. (2003) highlighting smoking, hypertension, and radiotherapy to be significantly associated with such complications [24]. Ensuring smoking cessation and blood pressure control before surgery, as well as a meticulous surgical technique minimising tissue trauma and advocating pressure garments may be a more effective tool than a postoperative drain [7].

In addition to preoperative optimization, the use of enhanced recovery after surgery (ERAS) programmes (ERP) throughout the patient pathway have been shown by a systematic review by Soterupulos et al. to reduce both the length of stay and analgesia requirements [25]. In addition to patient education, multi-modal analgesia, VTE and antibiotic prophylaxis, early drain removal by postoperative day 3 as advocated by many authors (Chan et al., Miranda et al.) coupled with active mobilisation are important parts of the ERAS programmes [1,14]. Avoiding drain use in the first instance by utilising PTS can further contribute to patient progress in terms of time to first walk, reducing the length of hospital stay and minimising the complications reported above.

There are several limitations to this review, with there only being four studies available in the literature meeting the criteria for analysis. In addition, all are retrospective in design with no defined variables by which patients were stratified into receiving either PTS or drain-assisted abdominal closure. Moreover, Nagarkar et al. included, in their data, patients who underwent abdominal closure with progressive tension sutures with the concomitant use of abdominal drains [5]. This has implications for our study in that it introduces progressive tension sutures as a confounding variable. The authors suggest inferring the results of this meta-analysis accounting for the inherent limitations of the study designs. High-quality, randomised trials are, therefore, recommended to further the current evidence base.

## Conclusions

The authors report the first meta-analysis within the literature comparing the use of drains in abdominal-based free-flap surgery for breast reconstruction with PTS closure and no drains. Alternative techniques, such as progressive tension sutures to assist in dead space reduction, can decrease the length of hospital stay, complication rate, and operative time. A limitation of this review includes the small number of studies as well as not all outcomes being reported homogeneously, with operative time being depicted by only two authors. Based on the current evidence, however, closure of the abdominal wounds in autologous breast reconstruction using abdominal flaps with PTS and no surgical drains appears to reduce the duration of hospital stay, reduce the incidence of complications, and result in a shorter operative time. The authors, however, suggest high-quality randomized control to add to the current evidence base.
